# Drying as an effective method to store soil samples for DNA-based microbial community analyses: a comparative study

**DOI:** 10.1038/s41598-023-50541-2

**Published:** 2024-01-19

**Authors:** Emily Smenderovac, Caroline Emilson, Karelle Rheault, Élodie Brazeau, Marie-Josée Morency, Patrick Gagné, Lisa Venier, Christine Martineau

**Affiliations:** Canadian Forest Service, Natural Resources Canada, Ottawa, Canada

**Keywords:** Biodiversity, Microbial ecology, Ecological genetics, Microbial ecology, Sequencing, DNA sequencing, Biological techniques, Isolation, separation and purification

## Abstract

Soil sampling for environmental DNA in remote and semi-remote locations is often limited due to logistical constraints surrounding sample preservation, including no or limited access to a freezer. Freezing at − 20 °C is a common DNA preservation strategy, however, other methods such as desiccation, ethanol or commercial preservatives are available as potential alternative DNA preservation methods for room temperature storage. In this study, we assessed five preservation methods (CD1 solution, 95% Ethanol, Dry & Dry silica gel packs, RNAlater, LifeGuard) along with freezing at − 20 °C, against immediate extraction on organic and mineral soils for up to three weeks of preservation. We assessed direct effects on DNA concentration and quality, and used DNA metabarcoding to assess effects on bacterial and fungal communities. Drying with Dry & Dry led to no significant differences from immediate extraction. RNAlater led to lower DNA concentrations, but effects on community structures were comparable to freezing. CD1, LifeGuard and Ethanol either caused immediate significant shifts in community structure, degradation of DNA quality or changes in diversity metrics. Overall, our study supports the use of drying with silica gel packs as a cost-effective, and easily applied method for the short-term storage at room temperature for DNA-based microbial community analyses.

## Introduction

While molecular ecology is widely recognized as a useful tool for studying soil biodiversity and health, there are significant barriers to implementing these techniques to samples collected in remote locations. One of these barriers is the ability to preserve samples in the field. There are many situations in environmental studies where sample preservation may be difficult, and where doing so via freezing or cooling may be infeasible, impractical or costly. These situations can include remote field work or international shipping of samples, which can limit international collaboration efforts. Validation of preservation strategies other than freezing and/or cooling can add flexibility to existing local sampling regimes, as well as enable soil sampling in situations where it was previously impossible. It also reduces the likelihood of sample losses due to shipping interruptions or delays^1^. These alternative preservation methods can simplify sampling, and thus increase sampling efficiency in more difficult to reach field locations.

Long term extreme low temperature storage of samples is widely thought to be the best practice for microbial ecology^2^, however freezer space can quickly become an issue and − 80 °C freezers are not available in every lab. Typically, − 20 °C to − 30 °C are more widely available and so, are more commonly used. Most researchers attempt to get samples into a freezer within 48 h of sampling after being held at cold temperatures (with ice, or freezer packs) in the field^3–5^. However, this is not always possible in remote locations, and maintaining samples at these temperatures during shipping requires use of dangerous goods such as dry ice. Freezing as a preservation method has been shown to cause some changes to specific genera abundances^6^, but little change to overall community structure^7,8^. Storing at temperatures ranging from 20 °C to 30 °C leads to shifts in microbial communities^9,10^, but there are some commercially available solutions (e.g., LifeGuard Soil Preservation Solution) that claim to maintain a sample community structure without freezing and others, such as RNAlater, that have been applied with mixed success at preserving community structures^5,11^. Some studies have even indicated that ethanol can be used to preserve samples at ambient temperatures (as is regularly employed in arthropod studies)^12^. Many of these solutions have been evaluated in other studies to see whether they are comparable to freezing, but their performance are inconsistent in complex matrices such as soil^5,11,13,14^. Desiccation (drying) has also been identified as a promising approach for preserving microbial communities in the short to long term^15^. These different preservation techniques utilize distinct (sometimes proprietary) mechanisms of inhibiting microbial activity and community change, ranging from damaging proteins and cell walls (ethanol), reducing water availability (desiccation, freezing), or inhibiting DNase and RNase activity (LifeGuard). Very few studies to date have compared the performance of techniques relying on various mechanisms simultaneously, and it is therefore still not clear what is the best soil preservation strategy for microbial ecology when freezing is not an option.

In this study, we evaluate the effectiveness of five different ambient temperature soil preservation approaches for microbial ecology, and compare them to optimal (i.e., DNA extraction on the day of sampling) and standard (i.e., freezing) approaches applied to two soil types (organic and mineral forest soil) from Quebec, Canada. We aim to answer the following: Is there a room temperature preservation method that can maintain soil microbial diversity and community structure? Does the best preservation method vary by sample type? How long can these preservation methods maintain sample diversity and community structure? This information will better inform field work planning for soil microbial ecology projects with temperate/boreal remote and semi-remote field work.

## Methods

### Sample collection and preservation

Soils for this experiment were collected at the Valcartier Forestry Research Station in Saint-Gabriel-de-Valcartier (Québec, Canada). Valcartier is a relatively flat landscape featuring a podzolic sandy loam soil on a fluvial plain deposit^16^. Two different soil types were collected: organic soil (0–7 cm depth) and, mineral soil (40–50 cm depth). Organic and mineral soils were collected using a shovel, homogenized by sieving in situ with a 6 mm sieve, and stored on ice in a plastic bag for 5 h. All samples were stored at 4 °C for no longer than 48 h upon arrival at the Laurentian Forestry Centre prior to the beginning of the experiment.

Soil samples were split and subjected to 7 preservation methods: Immediate extraction; Freezing in a − 30 °C Freezer (Freeze); CD1 solution of the DNeasy Powersoil Pro DNA extraction kit (QIAGEN, Valencia, CA, USA) (CD1); Dry & Dry (10 g Dry & Dry silica gel packs) (Dry); LifeGuard solution (QIAGEN) (LG); RNAlater solution (Invitrogen, Thermo Fisher) (RL); 95% Ethanol (EtOH). In order to simulate the application of these preservation methods in the field, liquid preservation solutions were prepared in advance by adding 1 mL of storage solution in 2 mL screw cap microtubes. Approximately 0.5 mL of soil was added to the 2 mL tube (corresponding to ~ 0.5 g organic soil, or ~ 1 g of mineral soil) and thoroughly mixed. This 2:1 solution-to-soil volume ratio was chosen to make the protocol suitable for field conditions, where a balance is not generally available, while also minimizing the total volume of reagents to reduce costs and space requirements. The Dry & Dry treatment was achieved by placing two silica gel packs in airtight plastic bags with one spoonful of soil (~ 8 g of organic soil or ~ 15 g mineral soil), again to mimic a realistic field sampling scenario. For the freezing preservation, a 15 mL tube filled with each soil type was stored at − 30 °C. All ambient temperature preservation methods (i.e., all except Immediate extraction and freezing) were stored in a 21 °C growth chamber and sampled at multiple time-points. Triplicate samples were prepared for each preservation method and time point. At time point one, two, and three weeks, one set of triplicate samples was removed from each of the room-temperature preservation methods. For the liquid preservation methods, each tube was centrifuged at max speed (15000×*g*) for 3 min, the supernatant was removed and the soil was transferred to sterile, absorbent paper to remove excess solution prior to to DNA extraction. For all preservation methods, soils were weighed into extraction tubes (100 mg for organic soil and 250 mg for mineral soil). DNA was extracted using the QIAGEN DNeasy Powersoil Pro kit for DNA extractions with the QIAcube system, following the manufacturer’s instructions. The initial cell disruption step was performed twice, using a TissueLyzer II (QIAGEN) set to an oscillation speed of 25 Hz for 5 min, as recommended by the manufacturer.

### DNA quantification and sequencing

DNA concentration of each DNA extract was measured using the Qubit™ dsDNA HS (or BR if concentration was too high) Assay Kit (Thermo Fisher) using 5 µL of sample and 195 µL of reagent. The readings were taken on the Qubit 3.0 fluorometer device (QIAGEN). DNA quality (260/280 ratio) was additionally measured using NanoDrop spectrophotography (Thermo Fisher). A 260/280 ratio of 1.8 is generally considered to be pure DNA, and variation away from this indicates contamination with RNA (if the ratio is higher) or indicates the presence of protein (when it is lower). Metabarcoding libraries targeting the 16S rRNA gene of bacteria and the ITS2 region of fungi were prepared following the procedure described in Rhealt et al.^17^, except that PCR reactions for the first amplification were set up by first mixing 25 μL of HotStarTaq Plus Master Mix, 19 µL RNase-Free Water (QIAGEN, Valencia, CA, USA), 0.5 μL of each 10 μM primer and 5 μL of gDNA at 5 ng/μL and annealing time was set to 45 s instead of 30 s. Additionally, indexed and purified amplicons were quantified using the Synergy™ Mx Microplate Reader (BioTek Instruments, Inc., Winooski, VT, USA) before pooling at equimolar concentration. Paired-end sequencing (2 × 250 bp) of the pools was carried out on an Illumina MiSeq sequencer at the Illumina Sequencing Platform, Nucleic Acids Solutions, Aquatic and Crop Resource Development, National Research Council Canada-Saskatoon. The Illumina sequence data generated in this study were deposited in the NCBI Sequence Read Archive and are available under the project number PRJNA982550 with the Sample Record numbers SRR24892048 to SRR2482149 for 16S sequences, and SRR24894315 to SRR24894416 for the ITS sequences.

### Bioinformatics

All bioinformatics analyses were performed using QIIME2 (version 2021.8)^18^. Raw demultiplexed sequences were denoised and dereplicated using the QIIME2 implementation of DADA2 (denoised-paired command algorithm)^19^, rare features (frequency less than 0.05% of the mean features frequency) were removed. The taxonomic assignment of ASVs was done using the SILVA 138 database for the 16S rRNA gene^20,21^ and the UNITE database (version 8.0) for the ITS2 region^22^. Only ASVs assigned to the kingdom Bacteria and Archaea (for the 16S rRNA gene) or Fungi (for the ITS2 region) were kept in the data set using the taxa filter-table command. Irrelevant taxa in 16S results (eukaryote, mitochondria, chloroplast) were removed using the same command.

### Reagent costs

Costs of each preservation agent were acquired from Amazon, ThermoFisher, QIAGEN and Fisher Scientific on February 27th, 2023, estimates of costs were all calculated in Canadian Dollars using the exchange rate on that day. Shipping and any additional costs were not assessed.

### Statistical analysis

All statistical analyses were performed in R v4.1.1^23^ using tidyverse v2.0.0^24^ for data transformations, while visualizations were performed with ggplot2 v3.4.1^25^. Preservation methods at each week of extraction were independently compared to immediate extraction using defined contrasts. For alpha diversity metrics, samples were rarefied to the 15th percentile of total sample reads. Shannon and inverse Simpson’s distances were calculated for bacterial relative abundance transformed data and ASV richness was calculated for both bacterial and fungal data. All diversity metrics were calculated with the diversity function of the vegan package and richness was calculated with the specnumber function of the vegan package v2.6–4^26^. A simple ANOVA model using the aov function in R was applied for DNA quality, DNA concentration, Shannon diversity, Inverse Simpson’s diversity and richness. Results were assessed in the context of Holm-adjusted^27^ p-values as well as unadjusted p-values, at an alpha of (p < 0.05) to provide different levels of confidence for observed effects. Community structure changes were tested with beta-dispersion and PERMANOVA (using the betadisper and adonis2 functions in vegan) on center-logged-ratio transformed datasets using Euclidean distance^26^, and visualized with ordination using PCA of Aitchison distances^28^. Individual ASV responses were assessed with the ancombc2 function in the ANCOMBC package v2.0.2^29^.

## Results

Liquids require 1 mL for sample preservation, and Dry & Dry uses two packs per sample.CD1 solution is included with QIAGEN DNeasy Powersoil Pro kit.

CD1, Dry & Dry silica packs and 95% Ethanol were the most cost-effective options, all being under $1CAD per sample, while LifeGuard and RNA later were more expensive (Table 1).

**Table 1:** Per-Sample costs of soil preservation for microbial ecology based on estimates acquired on 2023-02-27.

Treatment	Cost per unit	Unit size	Number of samples per unit	Additional Cost per sample ($CAD)	Supplier Information
CD1	N/A			0.00	QIAGEN
Dry & Dry	36.99	150 packs	75	0.49	Amazon
LifeGuard	3409.46	1000 mL botle	1000	3.41	QIAGEN, cat# 12868-1000
RNAlater	686	500 mL bottle	500	1.37	ThermoFisher, cat# AM7021
95% Ethanol	171.38	4000 mL bottle	4000	0.04	Fisher Scientific, cat# LC222054

DNA concentration ranged from 3 to 196 ng/uL in Organic soil, and 3–44 ng/uL in Mineral Soil. DNA Quality ranged from 1.12 to 2.01 in Organic soil, and 1.21 to 6.59 in Mineral soil.

### Preservation method performance by sample type

#### Organic soil

In organic soil, Dry & Dry was overall the most effective preservation method for both bacterial and fungal communities, with no significant changes from immediate extraction for all measured parameters (Table 2).

**Table 2:** Summary of the effect of preservation methods after two weeks of incubation compared to immediate extraction, in organic soil. Significant (p < 0.05) results are indicated with the effect size, and standard error in brackets and NS is used to represent non-significant results.

target	Parameter	Freezing	CD1	Dry & Dry	Ethanol	LifeGuard	RNALater
	DNA Concentration (ng/μL)	NS	NS	NS	− 160.91 (15.55)	− 120.07 (15.55)	− 116.73 (15.55)
	DNA Quality (260/280 ratio)	NS	NS	NS	− 0.33 (0.05)	NS	NS
Bac	Inv.Simpson (1/D)	NS	NS	NS	NS	NS	NS
Bac	richness (unique ASV)	NS	NS	NS	− 438.33 (157.82)	NS	NS
Bac	Shannon (H)	NS	NS	NS	NS	NS	NS
Fun	richness (unique ASV)	NS	− 106 (30.55)	NS	− 299.67 (30.55)	− 247 (30.55)	NS
Bac	PERMANOVA (R2)	NS	NS	NS	NS	NS	NS
Bac	beta-dispersion (Mean Square)	NS	NS	NS	NS	NS	NS
Fun	PERMANOVA (R2)	NS	NS	NS	NS	NS	NS
Fun	beta-dispersion (Mean Square)	NS	230.75	NS	NS	684.1	NS

**Figure 1:**
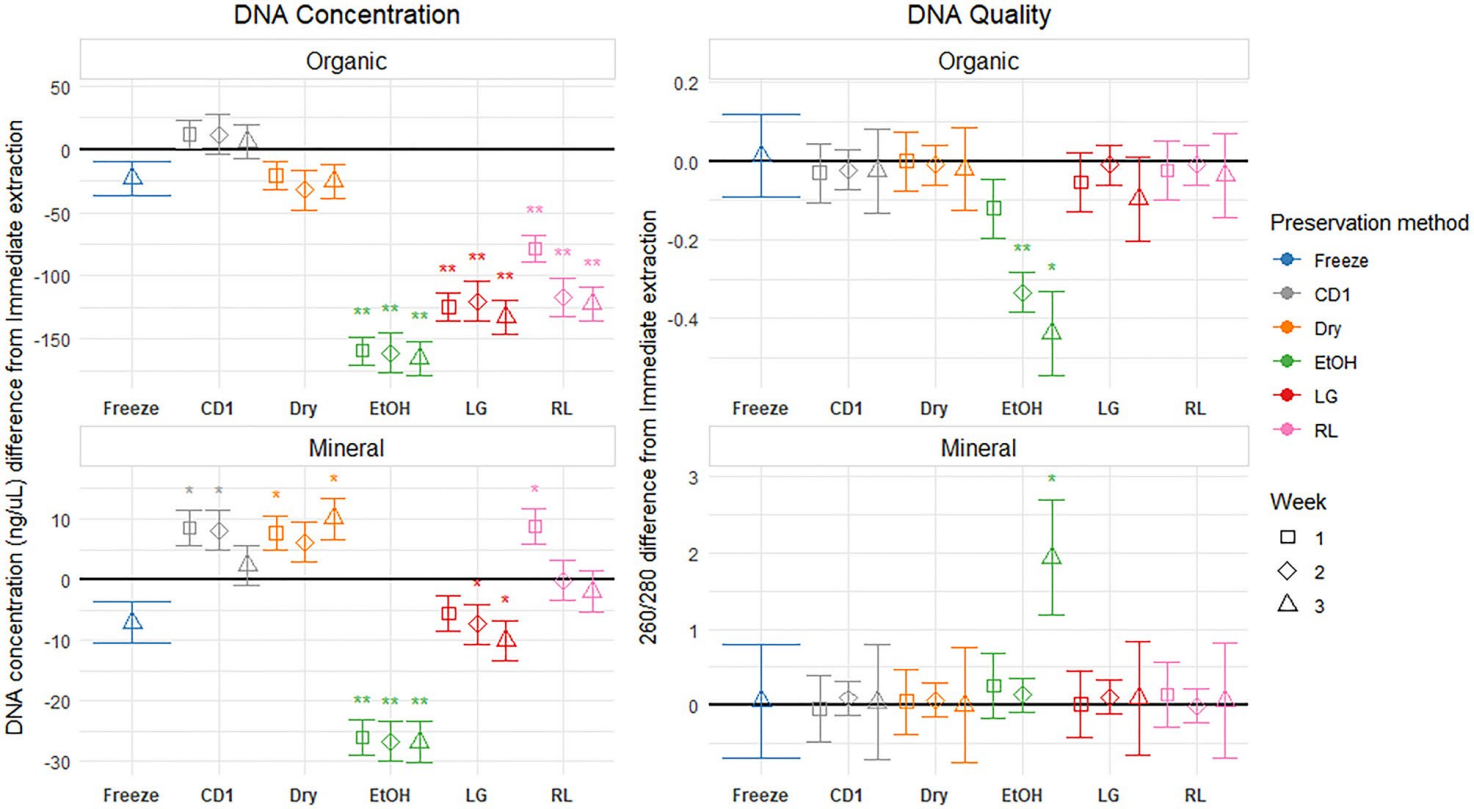
Preservation method effect size on DNA concentration (ng/μL) and DNA quality (260/280 ratio) compared to Immediate extraction. Points display the estimated effect (difference from immediate extraction, black line) introduced by the preservation method, and error bars represent the standard error of the estimate. For each preservation method, the incubation periods are displayed in order (week one to three) from left to right, except for Freezing, which was only tested on week three. Significant results are indicated with an asterisk; * represents significance at p < 0.05, and ** represents significance at a holm-adjusted p < 0.05.

For DNA extraction and quality, CD1 and Dry & Dry were comparable to Freezing, and the three methods effective for both bacterial and fungal communities as there were no significant changes to DNA concentration and quality compared to immediate extraction (Fig. 1, Table 2). The Lifeguard, RNAlater, and Ethanol preservation methods had significantly lower DNA yields compared to immediate extraction. The Ethanol preservation method also significantly reduced DNA concentration and reduced 260/280 ratio (Fig. 1, Table 2).

**Figure 2:**
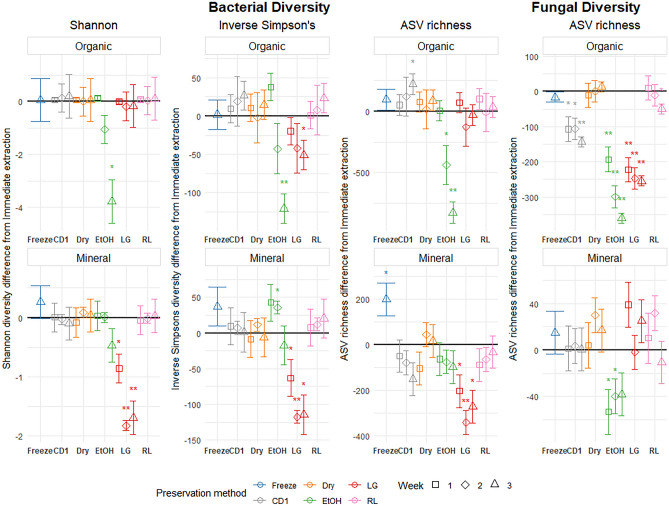
Preservation method effect sizes on bacterial Shannon diversity, inverse Simpson’s diversity, ASV richness and fungal ASV richness compared to Immediate extraction for forest mineral and organic soils. Points display the estimated effect (difference from immediate extraction) introduced by the preservation method, and error bars represent the standard error of the estimate. For each preservation method, the incubation periods are displayed in order (week one to three) from left to right, except for Freezing, which was only tested on week three. Significant results are indicated with an asterisk; * represents significance at p < 0.05, and ** represents significance at a holm-adjusted p < 0.05.

Ethanol and LifeGuard were the only preservation methods that significantly affected bacterial alpha diversity in organic soils (Fig. 2). Ethanol significantly decreased all bacterial diversity metrics after three weeks of incubation, and ASV richness after two weeks of incubation. LifeGuard only reduced inverse Simpson’s diversity after 3 weeks of incubation (Fig. 2).

In general, preservation methods had more effect on fungal ASV richness than bacterial alpha-diversity metrics. The CD1, Ethanol, RNAlater and LifeGuard preservation methods all caused significant decreases in fungal ASV richness in organic soils (Fig. 2, Table 2).

**Figure 3:**
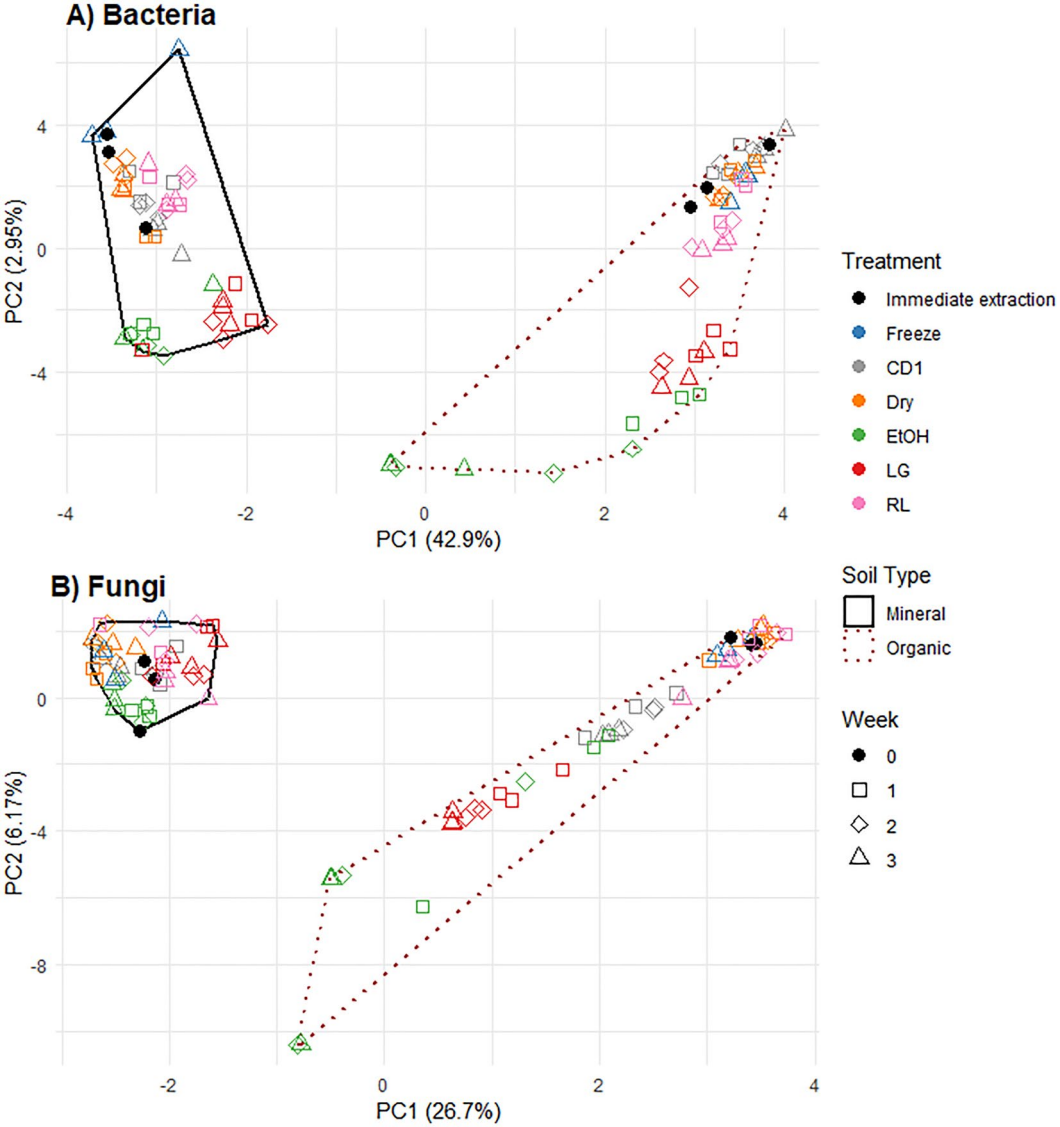
PCA of bacterial and fungal communites based on Aitchison distances for soil samples subjected to different storage conditions over time. (**A**) Ordination for bacterial communities for all preservation methods for the different soil types at all time points, (**B**) Ordination for fungal communities for all preservation methods for the different soil types at all time points.

**Figure 4:**
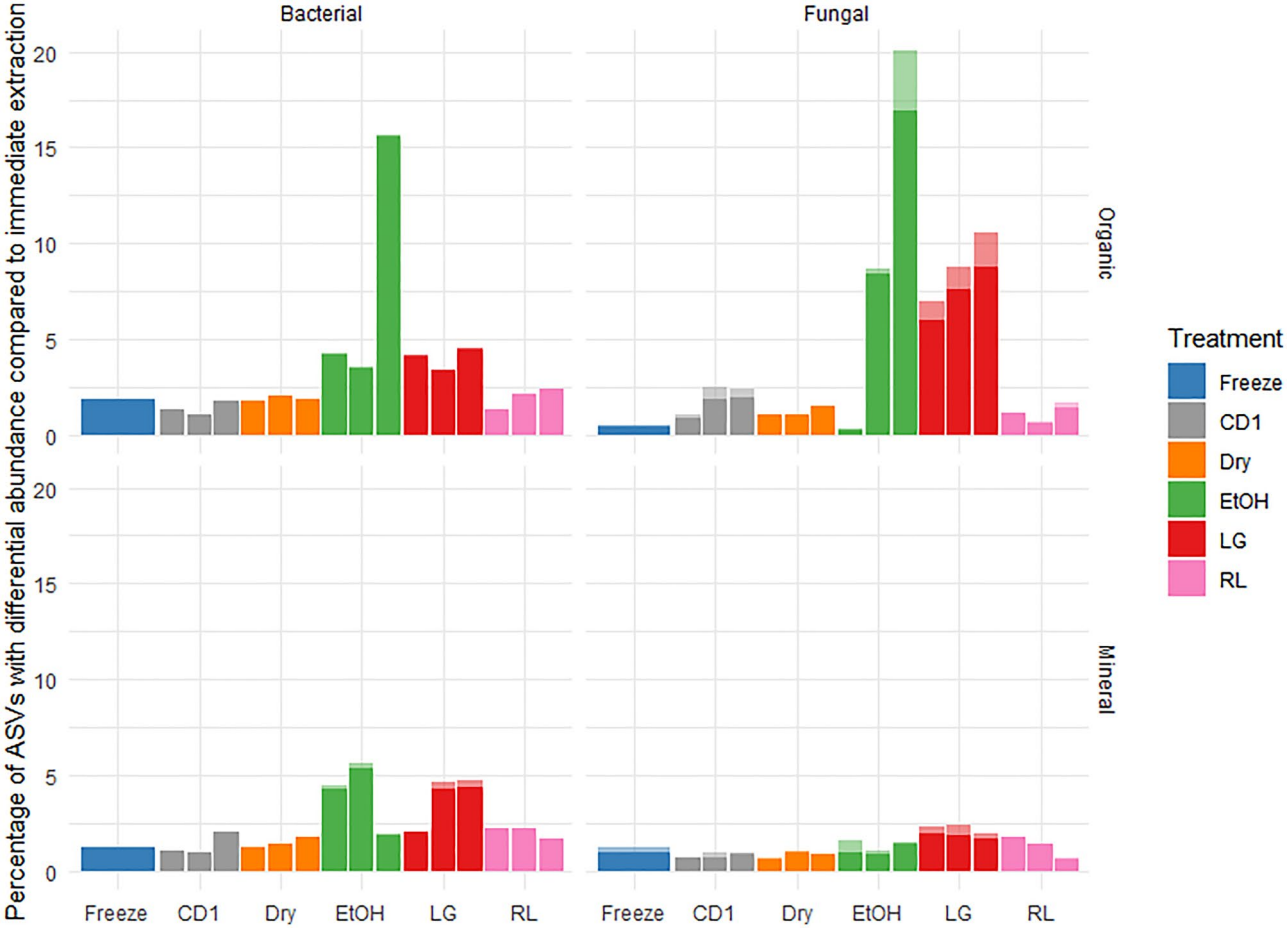
Percentage of ASVs with significant differential abundance compared to immediate extraction. Lighter colors represent the percentage of organisms that had an abundance above 1% in either immediate extraction, or the preservation method, the remaining dark portion of the bar represents organisms in the rare biosphere (< 1% relative abundance). Preservation methods are distinguished using colors and weeks are ordered 1–3 from left to right, except for Freezing, which was only tested on week three.

Although some visual groupings of samples by preservation treatment were observed in ordinations for both bacterial and fungal communities in organic soils, indicating some potential differences in community structure, especially for the Ethanol and Lifeguard treatments (Fig. 3), no significant changes to overall community structure were detected, aside from changes in fungal beta-dispersion (Table 2). Freezing, CD1, Dry & Dry and RNAlater had comparable percentages of bacterial ASVs which were differentially abundant compared to immediate extraction (Fig. 4). The remaining two preservation methods had the highest percentage of ASVs that were differentially abundant in organic soils compared to immediate extraction: LifeGuard (~ 4% of bacterial ASVs, ~ 10% of fungal ASVs) and Ethanol (~ 15% of bacterial ASVs, ~ 20% of fungal ASVs) (Fig. 4). Dry & Dry and RNAlater generally caused small (< 1% abundance) changes in generalist or potentially heat-tolerant organisms (e.g., *Bacillus*, *Acidothermus*, *Geoglossum*, *Mycobacterium*, *Penicillium*), while Ethanol and LifeGuard caused some large (> = 1% abundance) changes in organisms that may be of more ecological interest such as Ectomycorrhizae, wood and plant saprotrophs (e.g., *Hygrocybe*, *Hyphodontia*, *Melinomyces*, *Pestalotiopsis*). CD1 caused large changes in soil generalist organisms *Metapochonia*, *Ovicillum*, *Penicillium* and *Samsoniella*. Freezing resulted in a large change to one organism, identified as a *Geoglossum* (Supplemental Table S1).

#### Mineral soil

In mineral soil, Dry & Dry, CD1 and RNAlater were the most effective preservation methods for both bacterial and fungal communities, with no significant changes from immediate extraction aside from increased DNA concentration in CD1 solution (Table 3).

**Table 3:** Summary of the effect of preservation methods after two weeks of incubation compared to immediate extraction, in mineral soil. Significant (p < 0.05) results are indicated with the effect size including the standard error in brackets, and NS is used to represent non-significant results.

Target	Parameter	Freezing	CD1	Dry & Dry	Ethanol	LifeGuard	RNALater
	DNA Concentration (ng/μL)	NS	8.1 (3.29)	NS	− 26.78 (3.29)	− 7.37 (3.29)	NS
	DNA Quality (260/280 ratio)	NS	NS	NS	NS	NS	NS
Bac	Inv.Simpson (1/D)	NS	NS	NS	35.69 (9.01)	− 117.68 (9.01)	NS
Bac	richness (unique ASV)	199 (71.83)	NS	NS	NS	− 340.67 (52.4)	NS
Bac	Shannon (H)	NS	NS	NS	NS	− 1.83 (0.09)	NS
Fun	richness (unique ASV)	NS	NS	NS	− 40 (14.96)	NS	NS
Bac	PERMANOVA (R2)	NS	NS	NS	NS	NS	NS
Bac	beta-dispersion (Mean Square)	NS	NS	NS	NS	151.51	NS
Fun	PERMANOVA (R2)	NS	NS	NS	NS	NS	NS
Fun	beta-dispersion (Mean Square)	NS	NS	NS	NS	NS	NS

The Ethanol and Lifeguard preservation methods had negative effects on DNA yields in mineral soils. Ethanol had worse impacts than Lifeguard, and also impacted the DNA quality (Fig. 1). The other preservation methods either had no impact, or slightly increased DNA yields when compared to immediate extraction, while having no significant effect on DNA quality (Fig. 1, Table 3).

All bacterial alpha-diversity metrics were significantly decreased due to the LifeGuard preservation method in mineral soils. Ethanol and CD1 also had a significant impact on bacterial Shannon and inverse Simpson’s diversity after two weeks (Ethanol increase) and ASV richness after three weeks (CD1 decrease) (Fig. 2). For the fungal community, only the ethanol preservation had an effect on ASV richness: A significant decrease compared to immediate extraction was observed for fungal ASV richness in mineral soils (Fig. 2).

Visual grouping of samples by preservation treatment was also observed in ordinations for mineral soils, indicating some divergence of community structure (Fig. 3), but again, there were no significant community structural changes aside from the Ethanol and LifeGuard preservation methods having a significant effect on beta-dispersion of bacterial communities (Table 3). Freezing, CD1, Dry & Dry and RNAlater had comparable percentages of differentially abundant bacterial ASVs compared to immediate extraction, while higher percentages were detected for Ethanol and Lifeguard (Fig. 4). There were more differentially abundant fungal ASVs due to LifeGuard than freezing and a higher amount of abundant (> 1% abundant) fungal ASV responses to the Ethanol and Lifeguard treatments than the freezing treatment (Fig. 4). Dry & Dry caused small (< 1% abundance) changes in generalist organisms (e.g., *Bacillus*, *Penicilium*) and some larger changes a wood necrotroph, and a saptrotroph (*Scytalidium* and *Sympodiella*, respectively), CD1 also caused changes in a nectrotroph and saprotroph (*Scytalidium* and *Tolypocaldium*), while Ethanol and LifeGuard caused some large (> = 1% abundance) changes in organisms that may be of more ecological interest such as Ectomycorrhizae, wood and plant saprotrophs and some generalists (e.g., *Bacillus*, *Melinomyces*, *Penicillium*, *Trichoderma*), LifeGuard specifically had an increase of a sulfate reducing organism (*Desulfitobacterium*) which may have been responsible for the rotten-egg smell that was produced in the LifeGuard preserved samples. Freezing resulted in a large change to one organism but only by week three (*Sympodiella*) (Supplemental Table S2).

### Preservation stability over time

Generally, the effect of storage time on DNA quality and concentration was consistent through time for CD1, Dry & Dry, LifeGuard and RNAlater preservation (i.e., these treatments only caused an initial change, and they did not have additional change afterwards). Ethanol shifted from no significant difference from immediate extraction to a significant (p-value < 0.05) quality difference by week three (Fig. 1).

Differences in bacterial diversity estimates compared to immediate extraction were stable across time for all preservation methods aside from Ethanol. Ethanol preservation decreased bacterial diversity after one week (Fig. 2).

Fungal richness did not change much with incubation time in mineral and organic samples, generally maintaining a similar value to immediate extraction, or continuing to have significant decreases in diversity (Fig. 2).

While none of the preservation methods resulted in a significant difference in community structure for either bacterial or fungal communities, there was some indication in ordinations that Ethanol and LifeGuard preservation became more different from immediate extraction with longer incubation times in organic soil (Fig. 3). There were noticeable signs of microbial activity (i.e., rotten egg smell) that also corresponded with a change in *Desulfitobacterium* abundance in LifeGuard samples (e.g., rotten egg smell). There were also higher percentages of differentially abundant ASVs from immediate extraction for both fungi and bacteria in older ethanol treated organic soil samples (Fig. 4).

### Overall preservation method performance

Across all preservation methods and sample types, Dry & Dry and CD1 were the most similar to immediate extraction and had increased yields or were comparable with freezing in terms of the effects on DNA concentration or quality (Table 2, Table 3). Ethanol was the only preservation method in which the effects on quality and concentration of DNA extracted from samples resulted in poor sequencing; 9 ethanol-preserved samples had very poor recovery and sequencing results (less than 2500 reads/sample).

Only Freezing and LifeGuard had significant effects on bacterial alpha diversity metrics (Shannon diversity, inverse Simpson’s diversity or richness). Dry & Dry and RNAlater were the best methods to preserve fungal ASV richness with no significant difference from immediate extraction after a week of incubation at room temperature. For two weeks of incubation, Dry & Dry and RNAlater preservation also had the fewest significant changes from immediate extraction aside from freezing.

None of the preservation methods resulted in a significant difference in community structure for either bacteria or fungi when compared to immediate extraction, except for some significant changes in beta-dispersion in the CD1, Ethanol and LifeGuard preservation at week two (Tables 2, 3). In addition, the variance associated with the difference from immediate extraction was 10^5^ times lower than the differences between sample types for both bacteria and fungi (Fig. 3).

Freezing, Dry & Dry and RNAlater had comparable percentages of ASVs that with differential abundance compared to immediate extraction across both sample types. LifeGuard preservation method performed poorly across both sample types (Fig. 4).

## Discussion

### Which preservation methods can maintain soil microbial communities at room temperature?

Only two preservation methods were effective for both sample types assessed in this study: Dry & Dry and RNAlater both maintained DNA quality as well as community diversity and structure for bacteria and fungi in organic and mineral soils. There was lower DNA concentration with RNAlater preservation, but only in the organic soil type, potentially because it retained more of the high salt concentration solution, which could interfere with the DNA extraction process^30^ and this didn’t appear to affect the DNA quality or microbial community.

Storage of samples with Dry & Dry may be the most practical preservation method for organic and mineral soil samples. There are few complications involved with its use aside from keeping the silica dry: it is lightweight, takes up little space, and it is less costly than the commercially available liquid preservatives tested in this study. Additionally, Dry & Dry performed as good or better than any of the commercially available liquid preservation methods and even freezing. Though our results are specific to soils from one specific location, results from other studies corroborate that drying can be an effective preservation method for mineral and organic soil communities in soils from riparian, forest and grassland environments^9,15,31,32^. While microbial communities have been shown to respond to differences in drying-rewetting processes in the environment^33,34^, some researchers have suggested that this method is effective because microbial communities are subjected to regular drying cycles, and so there are normal dormancy adaptations that occur when drying stress is applied, preventing community shift^35,36^. Additionally, when the samples are dry enough, normal metabolic activities are slowed or stopped, preventing degradation of extracellular DNA^15,37,38^. Either way, this method may require further validation if working with soils with different properties (e.g., wet soils, or samples with low porosity or high clay content) as the preservation could be less effective if moisture is retained in the sample. Adding more silica gel packs to compensate for the additional moisture could be considered in that context.

Where liquid preservation is easily applied, RNAlater was effective (although not better than the Dry & Dry), even at a lower amount of solution than used by Schnecker et al.^5^, and the only downsides may be the lower DNA yields for organic soils and the logistical sampling constraints they introduce. Liquids, in general, are less desirable for remote field campaigns as they are difficult to manipulate (small tubes are used to reduce costs), allow for the collection of only small amounts of soil (so there is not as much back-up material), and require additional equipment and effort to use appropriately, which can be difficult in field settings. On the opposite spectrum, storage in LifeGuard at room temperature was not effective, leading to significant changes in alpha diversity and community structure when compared to immediate extraction. The 2:1 preservation solution to sample volume ratio used in this study was potentially too low for the LifeGuard solution to be effective, leading to a ratio of around 1 mL per gram for the mineral soil, vs the suggested 2–2.5 mL per gram of soil. However, changes in microbial communities were even larger in the organic soil despite the use of the recommended solution-to-sample ratio (i.e., around 2 mL per gram soil, indicating that this solution may be less performant than RNAlater as well as other methods used in this study (freezing, CD1, Dry & Dry)). These differences of efficacy between soil types support the need of including soil with various properties in studies evaluating the performance of different preservation methods. Increasing the volume of solution may have provided better results, but would have led to other issues, such as the need for larger containers to carry the samples, weight increases and maybe more importantly, higher costs. LifeGuard costs 3409.46 CAD per litre, which is approximately 8.53 CAD per sample at the suggested volume (2.5 mL per g soil) which can be prohibitive for large scale sampling campaigns. We did not explore the use of higher volumes because of the impracticality of the increased costs, when it was apparent that more cost effective and practical options exist (i.e., Dry & Dry). It is difficult to justify the increased cost of using LifeGuard when there is no guarantee that increased volumes would improve preservation. A study by Tatangelo et al.^39^, also reported that LifeGuard preservation led to community shifts when used on soil samples according to the manufacturer instructions. While we can only speculate on the reason underlying these shifts in communities observed with the LifeGuard preservation method or the effect of CD1 preservation on fungal diversity and composition because of the proprietary formulations of these solutions, it is likely that these changes are linked to some metabolic activity occurring during the incubation at room temperature. In the case of LifeGuard, this was further confirmed by the presence of a rotten egg smell suggestive of sulfate-reducing metabolism during the incubation at room temperature, and an increase in *Desulfitobacterium* abundance.

Ethanol was not an effective preservation solution in the short or longer term. Rissanen et al.^11^ also found DNA yields decreased with Ethanol and RNAlater preservation. Ethanol is generally toxic at high concentrations, and inhibits cell growth of soil microorganisms at as low as 10% concentration^40,41^, but can be metabolized by some organisms^42^. While other studies have investigated ethanol preservation and found it effective, they were usually only evaluating bacterial communities, while fungal richness was more impacted in our study^43^. The effect worsens over time, and resulted in large shifts in some highly abundant fungal ASVs. Since ethanol is toxic at high concentrations^41^, and because the organisms that shifted are not known to metabolize ethanol (e.g., the ectomycorrhizal genus *Tylospora* was a higher proportion of the community in mineral soil preserved in ethanol) it is unlikely that these changes would be linked to metabolic activity occurring in the tubes. The generally poor extraction from the ethanol-preserved samples could have been due to interactions between ethanol and DNA that may have also led to the differences in community composition observed with this preservation method. Ethanol can precipitate DNA while dissolving other inhibitory compounds such as humic acids and proteins^44^, which could be the possible mechanism of the DNA yield reductions.

The Dry & Dry or RNAlater preservation methods had no significant effects on community structure. Although there were differences in some specific ASVs with these two preservation methods, they were generally in the rare biosphere (< 1% abundance). These changes are minor compared to the changes in relative abundance of ectomycorrhiza, saprotrophs and other organisms that were biased by ethanol and LifeGuard preservation. While disturbance effects are generally lower than sample type differences in soil studies^45,46^, these can be detected in samples subjected to freezing at − 20 °C, so they will likely be detectable using Dry & Dry or RNAlater as preservatives as well. Freezing and Dry & Dry preservation may be more representative of normal seasonal processes than the other preservation methods tested in this study^36,47^. That is, though they may differ from natural processes in the speed and temperature, they should be more mechanistically similar than chemical preservation. Smenderovac et al.^3^, found that metabarcoding harvesting treatment responses were greater than any seasonal responses in boreal jack pine forest soils, so it may be that communities that experience these kinds of disturbances are resistant to the short-term changes brought upon by similar stresses. Silica desiccation has been applied to preserve water microbiomes^48^, and recently was found to be an effective preservative of soil fungal communities^49^. Guerrieri et al.^32^ found silica gel mostly effective for preserving bacterial and eukaryotic communities in soils, with the exception of changes in the rare biosphere, which was consistent with our findings. Moreover, though we did not test whether our soil storage prior to initiation of the experiment had an interaction with our preservation treatments, storing samples cooled in the field during the sampling period is a common approach that has been shown to have little effect on community structure^10,50,51^ and we believe this had little impact on the preservation, as our results largely matched those of other studies.

### How long can these methods maintain sample community structure?

Generally, the faster a sample is processed after sampling and subjected to DNA extraction the better, but the Dry & Dry treatment tested in this study effectively maintained communities at ambient temperature for up to three weeks without significant changes in DNA quality and quantity, diversity and community structure. Further work will be needed to determine if preservation of samples at ambient temperature could be extended past those three weeks, and if this stability over time is maintained for all soil types. Ivanova et al^52^ found that long-term (> 1 year) dry storage of soils had possible large effects on soil community structure, but several researchers have found that community structure with drying is particularly stable up to one month^9,15^, which was consistent with the three weeks of storage we found in our study. Until further work is done, samples should be frozen at − 20 °C or lower for time periods greater than three weeks, to avoid community shifts.

Stability of microbial communities over time was highly variable in the four liquid treatments tested in this study. RNAlater was the only liquid treatment that had stable communities for the full three weeks of the study, showing it was an effective preservative of these soils, despite the negative impact of this treatment on DNA quantity. The CD1 caused an immediate shift after one week that did not change much afterwards, while changes in community diversity metrics in weeks two and three suggest that microbial DNA is only temporarily stabilized by the Ethanol and LifeGuard preservation solutions. All treatments were tested at what is recognized as an ambient room temperature (21 °C), but it is important to consider that higher temperatures could reduce their effectiveness at stabilizing microbial communities over time. Ethanol results from the organic and mineral soils suggest that caution should be taken, as it can interact with DNA and/or inhibitors in unexpected ways^44^. Additionally, the general extraction and amplification issues encountered with the Ethanol preservation could be exacerbated within an extended time-frame and is therefore not recommended.

## Conclusion

There are many viable options for sample preservation in situations where immediate freezing may not be possible. Dry & Dry silica packs are logistically simple and cost effective, and were shown to be effective for preserving bacterial and fungal DNA in organic and mineral soils from this study. Sample preservation at room temperature with this method may be suitable up to three weeks for community structure and diversity assessments. The comparability of Dry & Dry to frozen samples suggest it is appropriate for organic or mineral soils. It is not suggested that ethanol preservation is used, as there seem to be chemical interactions with the sample matrix that interfere with DNA extraction. These preservation methods should be studied on a greater range of soil samples, and with more barcodes commonly used in soil biodiversity assessment (e.g., CO1, F230 used for the study of invertebrates) to understand the possible influences of different soil matrices on extraction effects, and whether particular adaptive histories affect responses to different preservation strategies. Future studies should aim to test whether different preservation methods can affect the interpretation of results or interact with the experimental design (e.g., climate gradients, forest management). This study cannot comment on the suitability of these preservation methods for metabolic, protein or enzyme analyses, which require different storage considerations to prevent protein degradation and/or cell viability. Further exploration of preservation approaches that allow for those analyses on remotely collected samples would be worthwhile. Overall, while there are some limitations and usage considerations, silica gel packs may be a useful tool that can either expand or enhance soil metagenomic sampling efforts in logistically challenging conditions in temperate and boreal forests.

### Supplementary Information

Supplementary Tables.

## Data Availability

All sequences used in this analysis are publicly available in the NCBI Sequence Read Archive and are available under the project number PRJNA982550[https://www.ncbi.nlm.nih.gov/bioproject/PRJNA982550] with the Sample Record numbers SRR24892048 through SRR24892149 for 16S sequences, and SRR24894315 through SRR24894416, for the ITS sequences. The RMarkdown and tabular data inputs used for creation of this manuscript are accessible on the https://github.com/Smendero/PRST repository.
