# Supplementary material for: Drying as an effective method to store soil samples for DNA-based microbial community analyses: a comparative study
Source: Sci Rep. 2024 Jan 19;14:1725. doi: 10.1038/s41598-023-50541-2 (PMC10798986; doi:10.1038/s41598-023-50541-2)
Supplement: Supplementary file 1 — Supplementary Tables. [file 41598_2023_50541_MOESM1_ESM.docx]

# Supplemental Tables

Supplemental Table 1: Overview of genuses with at least one preservation treatment that resulted in a large (>=1% relative abundance) change in an Organic soil sample. Direction and magnitude of change that occurred for each treatment are indicated with L (>1=% relative abundance change), s (<1% relative abundance change), - (decrease), + (increase). Treatments without any significant change were left blank.

| Type | Taxonomy | Week | Freeze | CD1 | Dry | EtOH | LG | RL |
| --- | --- | --- | --- | --- | --- | --- | --- | --- |
| Bac | Acidothermus | 1 |  |  | s+ | s+ | L+, s+ | s+ |
| Bac | Acidothermus | 2 |  |  | s+ |  | L+, s+ | s+ |
| Bac | Acidothermus | 3 | s+ | s+ |  | s- | s+, L+ | s+ |
| Bac | Bacillus | 1 |  |  |  | s+, L+ |  |  |
| Bac | Clostridium | 2 |  |  |  | L+ | s+ |  |
| Bac | Mycobacterium | 1 |  |  | s+ | L+, s+ | s+ | s+ |
| Bac | Psychrobacillus | 2 |  |  |  | L+ |  |  |
| Bac | RCP2 | 1 |  |  |  | L-, s- |  | s- |
| Fun | Apiotrichum | 1 |  |  |  |  | L+ |  |
| Fun | Apiotrichum | 2 |  |  |  |  | L+ |  |
| Fun | Apiotrichum | 3 |  |  |  | L- | L+ |  |
| Fun | Cenococcum | 3 |  |  |  | L-, s- | s- |  |
| Fun | Galerina | 3 |  |  |  | L- |  |  |
| Fun | Geoglossum | 1 |  |  | s- |  | L- |  |
| Fun | Geoglossum | 2 |  | s- | s- | s- | s-, L- | s- |
| Fun | Geoglossum | 3 | s-, L- | s- |  | s-, L- | s-, L- | s- |
| Fun | Hygrocybe | 1 |  |  |  |  | L- |  |
| Fun | Hygrocybe | 3 |  |  |  | L- | L- |  |
| Fun | Hyphodontia | 3 |  |  |  | L- |  |  |
| Fun | Mariannaea | 3 |  |  |  |  | L+ |  |
| Fun | Meliniomyces | 1 |  |  |  |  | L- |  |
| Fun | Meliniomyces | 2 |  |  |  |  | L- |  |
| Fun | Meliniomyces | 3 |  |  |  | L- | L- |  |
| Fun | Metapochonia | 2 |  | s+, L+ |  |  |  |  |
| Fun | Metapochonia | 3 |  | s+, L+ |  | s- |  |  |
| Fun | Mortierella | 2 |  |  |  | s- | L- |  |
| Fun | Mortierella | 3 |  |  |  | s-, L- | L- |  |
| Fun | Nectria | 3 |  |  |  |  | L+ |  |
| Fun | Ovicillium | 2 |  | L+ |  |  |  |  |
| Fun | Penicillium | 1 |  |  |  |  | L+, s+ |  |
| Fun | Penicillium | 2 |  | L+, s+ |  |  |  | L+ |
| Fun | Penicillium | 3 |  | L+, s+ |  | s-, L- |  | L+, s+ |
| Fun | Pestalotiopsis | 3 |  |  |  |  | L+ |  |
| Fun | Saitozyma | 3 |  |  |  | L-, s- |  |  |
| Fun | Samsoniella | 2 |  | L+ |  |  |  |  |
| Fun | Samsoniella | 3 |  | L+ | s+ |  |  |  |
| Fun | Trichoderma | 1 |  |  |  |  | L+ |  |
| Fun | Trichoderma | 2 |  |  |  | s- | s-, s+, L+ |  |
| Fun | Trichoderma | 3 |  |  |  | s-, L- | L+, s+ |  |

Supplemental Table 2: Overview of genuses with at least one preservation treatment that resulted in a large (>=1% relative abundance) change in a Mineral soil sample. Direction and magnitude of change that occurred for each treatment are indicated with L (>=1% relative abundance change), s (<1% relative abundance change), - (decrease), + (increase). Treatments without any significant change were left blank

| Type | Taxonomy | Week | Freeze | CD1 | Dry | EtOH | LG | RL |
| --- | --- | --- | --- | --- | --- | --- | --- | --- |
| Bac | Bacillus | 1 |  | s- | s+ | s+, L+ |  |  |
| Bac | Bacillus | 2 |  | s- | s+ | s+, L+ | L+, s- |  |
| Bac | Bacillus | 3 | s+ | s- | s+ | L+ |  |  |
| Bac | Desulfitobacterium | 3 |  |  |  |  | L+ |  |
| Bac | Paenibacillus | 2 |  |  |  | s+ | L+ |  |
| Bac | Paenibacillus | 3 | s+ |  |  |  | L+ |  |
| Fun | Geminibasidium | 1 |  |  |  | L+ |  |  |
| Fun | Meliniomyces | 3 |  |  |  |  | L+ |  |
| Fun | Penicillium | 3 |  | s+ | s+ |  |  | L+ |
| Fun | Scytalidium | 2 |  |  | L+ |  | L+ |  |
| Fun | Sympodiella | 1 |  |  | L- | L- | L- |  |
| Fun | Sympodiella | 2 |  | L- |  | L- | L- |  |
| Fun | Sympodiella | 3 | L- |  |  |  |  |  |
| Fun | Tolypocladium | 3 |  | L+, s- |  |  |  |  |
| Fun | Trichoderma | 1 |  |  |  |  | L+ |  |
| Fun | Venturia | 1 |  |  |  | L- |  |  |
| Fun | Venturia | 2 |  |  |  |  | L- |  |
